# Isoproterenol Increases RANKL Expression in a ATF4/NFATc1-Dependent Manner in Mouse Osteoblastic Cells

**DOI:** 10.3390/ijms18102204

**Published:** 2017-10-20

**Authors:** Kyunghwa Baek, Hyun-Jung Park, Jeong-Hwa Baek, Hyung-Ryong Kim

**Affiliations:** 1 Department of Pharmacology, College of Dentistry and Research Institute of Oral Science, Gangneung-Wonju National University, Gangwon-do 25457, Korea; daedanhae@gmail.com; 2 Department of Molecular Genetics, School of Dentistry and Dental Research Institute, Seoul National University, Seoul 08826, Korea; in2010@snu.ac.kr; 3 Graduate School, Daegu Gyeongbuk Institute of Science and Technology (DGIST), Daegu 42988, Korea

**Keywords:** isoproterenol, RANKL transcription, nuclear factor of activated T-cells cytoplasmic 1 (NFATc1), activating transcription factor 4 (ATF4), β-adrenergic receptor

## Abstract

Sympathetic nervous system stimulation-induced β-adrenergic signal transduction is known to induce bone loss and increase of osteoclast activity. Although isoproterenol, a nonspecific β-adrenergic receptor agonist, has been shown to increase receptor activator of NF-κB ligand (RANKL), the details of the regulatory mechanisms remain unclear. In the present study, we investigated the role of the nuclear factor of activated T-cells (NFAT) in isoproterenol-induced RANKL expression in C2C12 and in primary cultured mouse calvarial cells. Isoproterenol increased nuclear factor of activated T-cells cytoplasmic 1 (NFATc1) and RANKL expressions at both mRNA and protein levels and increased NFAT reporter activity. NFATc1 knockdown blocked isoproterenol-mediated RANKL expression. Isoproterenol also promoted cAMP response element-binding protein 1 (CREB1) and activating transcription factor 4 (ATF4) phosphorylation. Isoproterenol-mediated transcriptional activation of NFAT was blocked by protein kinase A (PKA) inhibitor H89. Isoproterenol-induced CREB1, ATF4, NFATc1, and RANKL expressions were suppressed by H89. Mutations in cAMP response element-like or NFAT-binding element suppressed isoproterenol-induced *RANKL* promoter activity. Chromatin immunoprecipitation analysis demonstrated that isoproterenol increased NFAT-binding and ATF4-binding activities on the mouse *RANKL* promoter, but did not increase CREB1-binding activity. Association of NFATc1 and ATF4 was not observed in a co-immunoprecipitation study. ATF4 knockdown suppressed isoproterenol-induced NFAT binding to the *RANKL* promoter, whereas NFATc1 knockdown did not suppress isoproterenol-induced ATF4 binding to the *RANKL* promoter. ATF4 knockdown suppressed isoproterenol-induced expressions of NFATc1 and RANKL. These results suggest that isoproterenol increases RANKL expression in an ATF4/NFATc1-dependent manner.

## 1. Introduction

A line of research has emerged detailing the role that the sympathetic nervous system (SNS) plays in bone-mass regulation. Chronic stress activates a defense mechanism via the release of catecholamines from sympathetic nerves and of glucocorticoids by the adrenal glands. The skeletal system is richly innervated by sympathetic nerve fibers that release norepinephrine as a neurotransmitter [1]. SNS regulation on bone remodeling and bone homeostasis has been well-demonstrated. SNS activation decreases osteoblast proliferation [2] and stimulates bone resorption by increasing receptor activator of NF-κB ligand (RANKL) expression [3]. Takeda et al. confirmed that osteoblasts express β-adrenergic receptors [2]. β_2_-adrenergic receptor-deficient mice showed a high bone mass phenotype [4]. The β-adrenergic receptor antagonist mitigated SNS activation-induced metaphyseal bone loss by attenuating declines in osteoblastic cell activity and by abolishing increases in osteoclast surfaces [5,6,7].

RANKL is a critical regulator of osteoclastogenesis. It is primarily produced by stromal cells/osteoblasts in bone tissue. In cooperation with colony-stimulating factor 1, RANKL induces osteoclast differentiation and stimulates its bone-resorbing activity [8,9]. Activation of the nuclear factor of activated T-cells (NFAT) transcription factors is known to be involved in bone resorption; in particular, nuclear factor of activated T-cells cytoplasmic 1 (NFATc1) is a pivotal transcription factor for osteoclast differentiation [10].

cAMP response element-binding protein (CREB) is a cellular transcription factor. There are several subtypes of CREBs (CREB1–5) and CREB-like proteins. CREB2 is also known as activating transcription factor 4 (ATF4). Once activated by phosphorylation, CREB protein binds alone or recruits other transcriptional coactivators to bind to cAMP response element (CRE) in the promoter region, thereby regulating various genes’ expression.

It has been demonstrated that downstream of cAMP/protein kinase A (PKA) activation, the activated CREB1 mediates PTH1R-induced RANKL expression by binding to multiple distal enhancers of the RANKL gene [11,12]. We previously reported that in the context of PTHrP-induced RANKL expression, cAMP/PKA and calcineurin/NFAT pathways are activated and, subsequently, CREB1 and NFAT cooperate to transactivate the mouse RANKL gene [13]. ATF4 is a critical transcription factor for osteoblast function and bone formation; however, a regulatory role in osteoclast differentiation or RANKL expression has not been fully studied.

To experimentally address that SNS activation stimulates bone resorption by increasing RANKL expression, a pharmacological model has been developed using β-adrenergic receptor agonists. Stimulation of the β-adrenergic receptor in osteoblasts by norepinephrine or isoproterenol, a pharmacological β-adrenergic receptor agonist, enhances osteoclastogenesis via upregulating RANKL expression [3]. In the context of SNS activation-induced RANKL expression, the role of the PKA/ATF4 signaling pathway has been demonstrated [3]. An in vivo study also showed that RANKL expression by mesenchymal stem cells increased after isoproterenol administration and decreased after administration of propranolol, a β-adrenergic receptor antagonist [14]. The β-adrenergic receptor signaling axis in the osteoblast lineage is thus of importance in RANKL regulation, which justifies the rationale for identifying the intracellular pathway of β-adrenergic receptor activation-induced RANKL expression in osteoblasts.

In the present study, we propose another transcriptional regulatory mechanism of β-adrenergic signaling-mediated RANKL expression, in which β-adrenergic signaling-induced NFATc1 upregulates RANKL transcription by directly binding to the mouse *RANKL* promoter.

## 2. Results

### 2.1. Isoproterenol Induces Expression Levels and Transcriptional Activity of NFATc1, Which Is Necessary for Isoproterenol-Induced RANKL Expression

**Figure 1:**
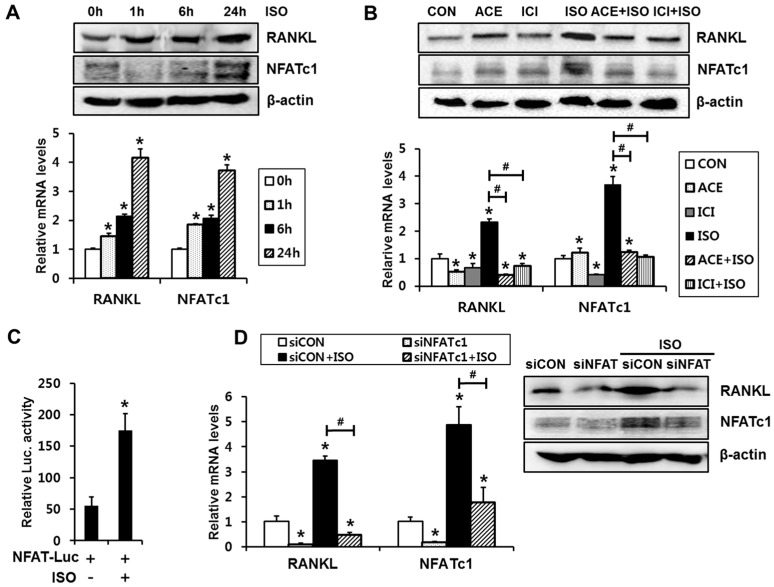
Isoproterenol induces expression levels and transcriptional activity of NFATc1, which is necessary for isoproterenol-induced RANKL expression. (**A**) Isoproterenol (ISO) increased the expression levels of RANKL and NFATc1. C2C12 cells were incubated in the presence of ISO at a concentration of 1 µM for the indicated time periods, followed by quantitative RT-PCR and Western blot analyses. * *p* < 0.05, compared to 0 h. (**B**) Both β_1_- and β_2_-adrenergic receptor subtypes were involved in ISO-induced RANKL and NFATc1 expression. C2C12 cells were treated with ISO for 24 h with the pretreatment of the selective β_1_- or β_2_-adrenergic receptor inhibitors (acebutolol (ACE), 1 µM; ICI-118551 (ICI), 1 µM), followed by RT-PCR and Western blot analysis. * *p* < 0.05, compared to CON. ^#^ *p* < 0.05, compared to ISO alone. (**C**) ISO-enhanced transcriptional activity of NFATc1. C2C12 cells were transfected with a reporter plasmid containing a NFAT response element (NFAT-Luc) and incubated for 24 h with or without ISO. * *p* < 0.05, compared to NFAT-Luc alone. (**D**) The knockdown of NFATc1 inhibited ISO-induced RANKL expression. C2C12 cells were transiently transfected with NFATc1 siRNA (siNFATc1) or non-targeting control siRNA (siCON) and incubated for 24 h in the presence or absence of ISO. Statistical significance was determined using one way ANOVA. * p < 0.05, compared to vehicle-treated siCON. ^#^ *p* < 0.05, compared to ISO-treated siCON. For all experiments, the quantitative data were presented as the mean ± SD.

First, we examined whether isoproterenol induces RANKL expressions and NFATc1 activation in C2C12 cells. The cells were incubated in the presence of 1 µM isoproterenol for 1, 6, and 24 h. Isoproterenol increased mRNA and protein levels of RANKL expression, which peaked at 24 h. (Figure 1A). In addition to RANKL, a significant increase in NFATc1 expression with isoproterenol was observed, and the expression level of NFATc1 was also highest at 24 h (Figure 1A). Thus, we used the 24 h time point to perform the following experiments.

To confirm the relative importance of β-adrenergic receptor subtypes, we examined the effect of acebutolol (ACE; β_1_-adrenergic receptor blocker) and ICI-118551 (ICI; β_2_-adrenergic receptor blocker) on isoproterenol-induced RANKL expression. The induction of *RANKL* expression by isoproterenol was significantly mitigated by treatment with ACE (1 µM) or ICI (1 µM) (Figure 1B). Similar to the effect on RANKL expression, expression levels of NFATc1 were also attenuated by both ACE and ICI (Figure 1B). These data confirmed that isoproterenol induced RANKL and NFATc1 expression in a β_1_/β_2_-adrenergic receptor-dependent manner, suggesting that both the β_1_- and β_2_-adrenergic receptors are involved in SNS activation-induced RANKL expression in osteoblastic cells.

Since the above data showed that activation of β-adrenergic receptor increases NFATc1 expression levels, we next examined whether isoproterenol enhances transcriptional activity of NFAT using a reporter plasmid containing a NFAT-responsive element [15]. Isoproterenol treatment for 24 h significantly induced NFAT reporter activity (Figure 1C). Since isoproterenol increased NFATc1 expression and transcriptional activity, we next examined the relative role of NFATc1 in the isoproterenol-induced RANKL expression by inducing NFATc1 knockdown. Transient transfection of NFATc1 siRNA decreased the mRNA and protein levels of basal and isoproterenol-induced NFATc1 (Figure 1D). NFATc1 knockdown also blocked the isoproterenol-induced upregulation of RANKL expression (Figure 1D). These results indicate that activation of NFATc1 is required for isoproterenol-induced RANKL expression.

### 2.2. PKA Signaling Is Involved in Isoproterenol-Induced NFATc1 Activation and Expression

**Figure 2:**
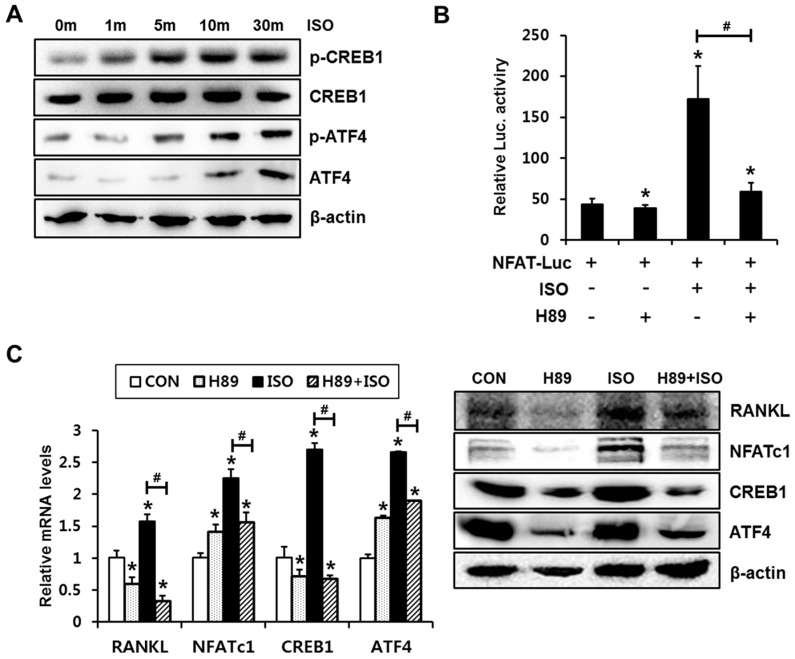
PKA signaling is involved in ISO-induced NFATc1 activation and expression in C2C12 cells. (**A**) ISO induced CREB1 and ATF4 phosphorylation. C2C12 cells were incubated with ISO for the indicated time periods, followed by Western blot analysis. (**B**) The activation of the PKA increased NFAT transcriptional activity. C2C12 cells were transfected with the NFAT reporter plasmid and were incubated for 24 h in the presence or absence of ISO and PKA inhibitor, H89 (20 µM). * *p* < 0.05, compared to vehicle control. *^#^ p* < 0.05, compared to the indicated pair. (**C**) ISO-induced RANKL, NFATc1, CREB1, and ATF4 expression was suppressed by inhibition of the PKA activity. C2C12 cells were incubated in the presence or absence of ISO and H89 for 24 h, followed by RT-PCR and Western blot analyses. Statistical significance was determined using one way ANOVA. * p < 0.05, compared to CON. *^#^ p* < 0.05, compared to ISO alone.

When stimulated, osteoblast β-adrenergic receptor primarily binds to the G stimulatory (Gs) protein. The Gα subunit activates adenyl cyclase, generating the second messenger cAMP; elevated cAMP levels activate the PKA signaling pathway [16]. We previously reported that cAMP/PKA signaling is involved in the PTHrP-induced activation of NFAT in C2C12 cells [13]. Thus, we next examined whether the cAMP/PKA pathway is also relevant to isoproterenol-mediated induction of RANKL/NFATc1 expression and NFATc1 activation. To explore whether isoproterenol induces PKA activation in C2C12 cells, CREB phosphorylation levels were assessed. Isoproterenol increased CREB1 and ATF4 phosphorylation (Figure 2A). Next, C2C12 cells were pretreated with a H89 for 1 h, then incubated with isoproterenol (1 µM) for 24 h. We then examined the effect on isoproterenol-induced NFATc1 activation and RANKL expression. Luciferase reporter assay showed that isoproterenol-induced transcriptional activation of NFAT was blocked by H89 (Figure 2B). Isoproterenol-induced CREB1, ATF4, NFATc1, and RANKL expressions were significantly suppressed by H89 at both mRNA and protein levels (Figure 2C). These results suggest that the isoproterenol-induced NFATc1 activation and RANKL/NFATc1 expression is PKA signaling pathway-dependent.

### 2.3. Binding of Both NFATc1 and ATF4 to the RANKL Promoter Is Necessary for Transactivation of RANKL Gene

**Figure 3:**
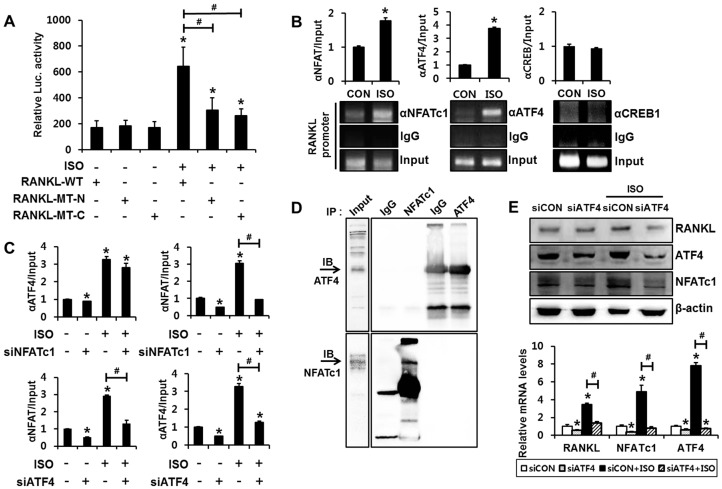
Binding of both NFATc1 and ATF4 to the *RANKL* promoter is necessary for transactivation of RANKL gene. (**A**) ISO increased *RANKL* promoter-reporter activity in a NFAT-binding element- and CRE-like element-dependent manner. C2C12 cells were transfected with the indicated *RANKL* promoter reporters and incubated for 24 h in the presence or absence of ISO, followed by the luciferase assay. Data were presented as the firefly luciferase activity relative to *Renilla* activity. * p < 0.05, compared to RANKL-WT without ISO. RANKL-WT: luciferase reporter containing approximately 2 kb of mouse *RANKL* promoter; RANKL-MT-N: a reporter with mutated sequences in NFAT binding element (−941 to −936 bp); RANKL-MT-C: a reporter with insertion of mutations in CRE-like element (−1093 to −1086 bp). (**B**) ISO increased the binding activity of NFATc1 and ATF4, but not CREB1, to the mouse *RANKL* promoter. C2C12 cells were incubated for 24 h with ISO and the chromatin immunoprecipitation assay was performed with antibodies to NFATc1, ATF4, CREB1, and control IgG. The *RANKL* promoter region containing the NFAT binding element or CRE-like element was then amplified. The quantitative chromatin immunoprecipitation data were normalized to the input and were presented as the relative values to CON. * p < 0.05, compared to CON. (**C**) The knockdown of NFATc1 blocked ISO-induced NFATc1 binding, but did not affect ISO-induced binding of ATF4 to the *RANKL* promoter (upper panels). The knockdown of ATF4 blocked not only ISO-induced ATF4 binding but also NFATc1 binding, to the *RANKL* promoter (lower panels). C2C12 cells were transfected with the indicated siRNAs and incubated for 24 h in the presence or absence of ISO, followed by chromatin immunoprecipitation assays. * p < 0.05, compared to vehicle-treated siCON. *^#^ p* < 0.05, compared to ISO-treated siCON. (**D**) NFATc1 was not co-immunoprecipitated with ATF4. NFATc1 and ATF4 were overexpressed in C2C12 cells, and immunoprecipitation (IP) was performed with the indicated antibodies, followed by immunoblotting (IB). Arrows indicate the bands for ATF4 (upper panel) and NFATc1 (lower panel). (**E**) The knockdown of ATF4 blocked ISO-induced RANKL and NFATc1 expression. C2C12 cells were transiently transfected with ATF4 siRNA (siATF4) or non-targeting siRNA (siCON) and incubated for 24 h in the presence or absence of ISO, followed by RT-PCR and Western blot analyses. * p < 0.05, compared to siCON. *^#^ p* < 0.05, compared to the indicated pair. Statistical significance was determined using one-way ANOVA.

We next explored whether the NFAT-binding element or CRE-like element in the mouse *RANKL* promoter is functional to isoproterenol-induced RANKL expression. Luciferase reporter assays were conducted using the *RANKL* promoter-reporter construct that contains approximately 2 kb of the DNA sequence located upstream of the transcription start site of the mouse *RANKL* gene [17]. An approximately three-fold increase in luciferase activity with the RANKL-WT reporter was observed with isoproterenol treatment; however, the insertion of mutations into the NFAT-binding element (−941 to −936 bp) or in the CRE-like element (−1093 to −1086 bp) prevented isoproterenol from significantly increasing reporter activity (Figure 3A), suggesting that direct binding of NFATc1 and CREB is also necessary for isoproterenol-induced RANKL transcription.

Next, we examined whether isoproterenol induces the binding of NFATc1 to the *RANKL* promoter. ChIP assay was performed to observe the direct binding of NFATc1 to the *RANKL* promoter. PCR amplification of the DNA region containing the NFATc1-binding element revealed that isoproterenol enhanced NFATc1 binding to the *RANKL* promoter (Figure 3B). Since isoproterenol enhanced CREB1 and ATF4 phosphorylation, we also examined whether isoproterenol regulates CREB1 and ATF4 binding to the *RANKL* promoter. Significant induction of ATF4 binding by isoproterenol was observed in the samples immunoprecipitated with ATF4 antibody (Figure 3B). However, the ChIP assay using the CREB1 antibody did not show a significant induction of CREB1 binding to the *RANKL* promoter by isoproterenol treatment. These results suggest that isoproterenol induces the binding of NFATc1 and ATF4, but not CREB1, to their cognate binding domain in the *RANKL* promoter. Furthermore, ChIP assays demonstrated that NFATc1 knockdown significantly suppressed isoproterenol-induced binding of NFATc1 to the *RANKL* promoter (Figure 3C). In a similar manner, ATF4 knockdown significantly suppressed isoproterenol-induced binding of ATF4 to the *RANKL* promoter. Notably, ATF4 knockdown significantly suppressed isoproterenol-induced binding of NFATc1 to the *RANKL* promoter, whereas NFATc1 knockdown did not affect isoproterenol-induced binding of ATF4 to the *RANKL* promoter (Figure 3C).

The CRE-like element (−1093 to −1086 bp) and the NFAT-binding element (−941 to −936 bp) in the murine *RANKL* promoter are located close to each another. A previous study demonstrated that CREB1 and NFAT cooperate to bind and transactivate the *RANKL* promoter in mouse osteoblastic cells [13]. Therefore, we next performed co-immunoprecipitation assays to examine whether protein-protein interactions occur between ATF4 and NFATc1. The ATF4 band was not detected in the sample immunoprecipitated with NFATc1 antibody, and NFATc1 band was not detected in the anti- ATF4-immunoprecipitated sample either, which indicates that ATF4 does not interact with the NFATc1 protein (Figure 3D).

To explore the underlying mechanism in suppression of NFATc1 binding to the *RANKL* promoter by ATF4 knockdown, NFATc1 and RANKL expressions were assessed in the context of ATF4 knockdown. ATF4 knockdown significantly suppressed isoproterenol-induced expressions of NFATc1 and RANKL (Figure 3E). This suggests that isoproterenol-induced ATF4 activation increases NFATc1 expression and that increased NFATc1 directly binds to the promoter region and contributes to RANKL gene transactivation.

### 2.4. Isoproterenol Induced the Binding of NFATc1 and ATF4 to the RANKL Promoter in Primary Cultured Mouse Calvarial Cells

**Figure 4:**
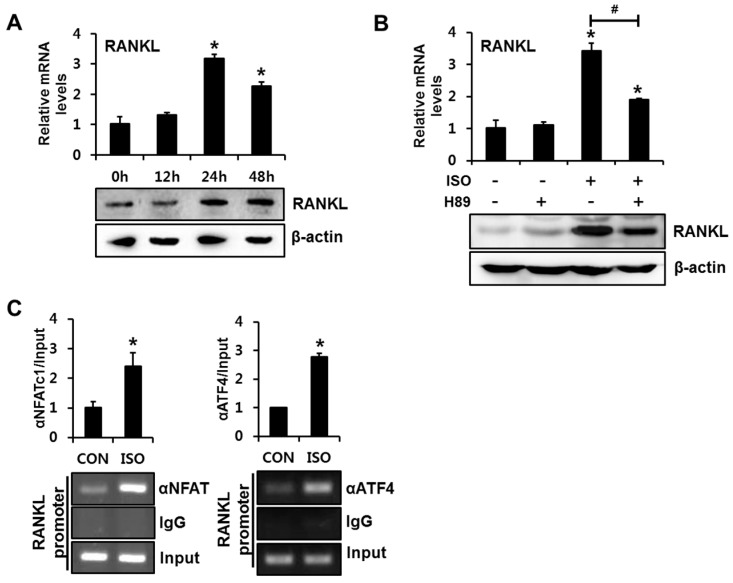
Isoproterenol-induced binding of NFATc1 and ATF4 to the RANKL promoter is also observed in primary cultured mouse calvarial cells. (**A**) ISO enhanced the RANKL expression levels. Calvarial cells were incubated in the presence of ISO for the indicated time periods, followed by RT-PCR and Western blot analysis. * p < 0.05, compared to 0 h. (**B**) ISO-induced RANKL expression was suppressed by inhibition of the PKA pathway. Calvarial cells were incubated in the presence or absence of ISO and H89 (20 µM) for 24 h, followed by RT-PCR and Western blot analyses. * p < 0.05, compared to CON. *^#^ p* < 0.05, compared to ISO alone. (**C**) ISO increased the binding of NFATc1 and ATF4 to the mouse RANKL promoter. Calvarial cells were incubated for 24 h with ISO and the chromatin immunoprecipitation assay was performed with the indicated antibodies. Statistical significance was determined using one-way ANOVA. * p < 0.05, compared to CON.

We next confirmed the involvement of NFATc1 and ATF4 in isoproterenol-induced RANKL transactivation in primary cultured mouse calvarial (MC) cells. Consistent with the results observed in C2C12 cells, isoproterenol induced RANKL expression in both mRNA and protein levels after 24 h of incubation (Figure 4A). Inhibition of PKA activity with H89 blocked isoproterenol-induced RANKL mRNA and protein expression (Figure 4B). ChIP assays with NFATc1 or ATF4 antibody demonstrated that isoproterenol increased NFATc1 and ATF4 binding to their cognate binding elements in *RANKL* promoter (Figure 4C). These results further support the hypothesis that isoproterenol enhances RANKL expression via PKA-dependent activation of ATF4 and NFATc1 and subsequent binding of these transcription factors to the *RANKL* promoter.

## 3. Discussion

In the present study, we demonstrated that, in isoproterenol-stimulated cells, NFATc1 binds to and transactivates the *RANKL* promoter. We provided the evidence supporting the functional relevance of the NFAT binding in the *RANKL* promoter in isoproterenol-induced RANKL expression as follows: (i) isoproterenol enhances expression levels and transcriptional activities of NFATc1 in a PKA-dependent manner; (ii) mutations in the NFAT-binding element suppressed isoproterenol-induced *RANKL* promoter activity; (iii) NFATc1 knockdown abolished isoproterenol-induced RANKL expression; and (iv) ChIP assays with NFATc1 antibody showed isoproterenol induces NFATc1 binding to the *RANKL* promoter.

Several mechanistic pathways have been demonstrated to elucidate the role NFATc1 in RANKL expression. We previously reported that PGE_2_-induced activation of NFATc1 is required in the context of TNFα-induced RANKL expression in mouse osteoblasts [18]. In the case of PTHrP-induced RANKL expression in mouse osteoblastic cells, it has been shown that NFAT signaling, in cooperation with CREB1 signaling, is required [13]. Lee et al. demonstrated that high extracellular calcium increases expression of RANKL via activation of the calcineurin/NFAT pathway in osteoblasts [17]. In addition to the osteoblast lineage, the role of NFAT in RANKL expression in oral squamous cell carcinoma (OSCC) cells has been reported. Yuvaraj et al. demonstrated that NFATc3 is a downstream target of the CXCL13/CXCR5 axis to stimulate RANKL expression in OSCC cells [19].

It is of significance that our results demonstrated that NFAT also plays a crucial role in the context of isoproterenol-induced RANKL expression in mouse osteoblastic cells. In the context of PTHrP-induced or TNFα-induced RANKL expression, CREB1 and NFATc1 binding and their cooperation to transactivate RANKL promoter has been reported [13,18], while in the context of SNS activation-induced RANKL expression, it has been demonstrated that ATF4 mediates isoproterenol-induced RANKL expression [3]. In the present study, we also demonstrated that ATF4 binds to the *RANKL* promoter and that mutations in CRE-like element block isoproterenol induction of *RANKL* promoter activity. Although CREB1 was also activated by isoproterenol treatment in C2C12 cells, binding of CREB1 to the CRE-like element was not induced by isoproterenol in this study. It is not clear how binding of ATF4, but not that of CREB1, to the CRE-like element is induced in the context of β-adrenergic receptor activation.

Our study results revealed the axis of ATF4 and NFATc1 in the isoproterenol- induced RANKL expression. In the present study, ATF4 knockdown suppressed isoproterenol-induced binding of NFATc1 to the *RANKL* promoter, whereas NFATc1 knockdown did not suppress isoproterenol-induced binding of ATF4 to the *RANKL* promoter. NFATc1 expression was dramatically decreased by ATF4 knockdown in C2C12 cells. These data imply that ATF4, in the upstream axis, activates NFATc1 gene transcription. These data are similar to the previous report showing that ATF4 acts as an upstream activator of the NFATc1 in osteoclast lineage cells [20].

We previously showed that cAMP/PKA signaling activation increases NFAT transcriptional activity through MEK/ERK activation in the context of PTHrP induction of RANKL expression [13]. In the present study, U0126, an inhibitor of MEK1/2, significantly suppressed isoproterenol-induced NFAT transcriptional activity and its activation on RANKL transcription (data not shown). These data suggest that β-adrenergic signaling-induced PKA activation stimulates not only NFAT transcriptional activity but also NFAT expression through ATF4 activation.

Thus, SNS activation stimulates bone resorption, at least through the following pathway. β-adrenergic signal transduction increases RANKL expression in ATF4-NFAT axis-dependent manners, and NFAT directly binds and transactivates the *RANKL* promoter in mouse osteoblastic cells. The results of the present study establish a direct and important role for NFAT in regulating isoproterenol-induced RANKL expression. Our data suggest that NFAT inhibition in osteoblasts may contribute to suppress bone resorption by downregulating RANKL expression.

Previous studies have shown that increases in osteoclast number and bone resorption under SNS-activated conditions such as mechanical unloading [21] and ovariectomy [2,22,23] are diminished by β-adrenergic signaling antagonists. The mechanisms underlying SNS activation-induced bone loss are complex and interrelated. However, β-adrenergic signaling pathways in osteoblasts in the context of SNS activation, which ultimately regulates the development of osteoclasts, have not been fully studied. Therefore, it is of significance our study elucidates the β-adrenergic signal transduction in osteoblasts increases RANKL expression in an ATF4/NFATc1-dependent manner. These findings provide new insight into the molecular basis of SNS activation-induced osteoclastogenesis and a rationale for development of novel therapeutic agents for bone loss associated with SNS activation.

## 4. Materials and Methods

### 4.1. Reagents and Antibodies

Isoproterenol, acebutolol, ICI-118551, and H89 were purchased from Sigma (St. Louis, MO, USA). Antibodies to RANKL, NFATc1, ATF4, and β-actin were obtained from Santa Cruz Biotechnology (Dallas, TX, USA), and horseradish peroxidase (HRP)-conjugated secondary antibodies were purchased from Thermo Fisher Scientific (Waltham, MA, USA). CREB1 and phospho-CREB1 antibodies were obtained from Cell Signaling Technology (Danvers, MA, USA), and phospho-ATF4 antibody was purchased from MyBioSource, Inc. (San Diego, CA, USA).

### 4.2. Cell Culture

C2C12 cells were cultured in Dulbecco’s modified Eagle’s medium (DMEM) supplemented with 10% fetal bovine serum (FBS) and antibiotics (100 µg/mL of penicillin/streptomycin).

We isolated MC cells from neonatal ICR mice calvaria and cultured in α-minimum essential medium (α-MEM) containing 10% FBS and antibiotics, as previously published [24]. For preparation of MC cells, we weaned animals in mouse gang cages following IACUC policies. The animal study was approved by the Special Committee on Animal Welfare, Seoul National University (approval no. SNU-20140228-1-5). DMEM, α-MEM, and FBS were purchased from Hyclone (Walkersville, MD, USA).

### 4.3. Plasmid Construction

Construction of the reporter plasmid containing an NFAT-responsive element has been reported previously [25]. A luciferase reporter containing a DNA sequence region −2174 to +1 bp of the mouse *RANKL* promoter (RANKL-WT) and a function-defective mutant reporter (RANKL-MT-N) containing mutations in the NFAT-binding site (−941 to −936 bp; GGAAAA → Gc*tt*AA) have been described previously [17]. Another function-defective *RANKL* promoter-reporter (RANKL-MT-C) containing mutations in the CRE-like element (−1093 to −1086 bp; TGAGGTCA → TGAGG*agg*) was also used in the present study [13,26].

### 4.4. Reverse Transcription-Polymerase Chain Reaction

We performed quantitative RT-PCR analysis to observe RANKL, NFATc1, CREB1, and ATF4 mRNA levels. Total RNA was prepared using easy-BLUE RNA extraction reagents (iNtRON Biotechnology, Sungnam, Korea). The first-strand cDNA was synthesized with AccuPower RT PreMix (Bioneer, Daejeon, Korea) and used for PCR amplification with SYBR premix EX Taq (Takara, Otsu, Japan). The primer sequences for real-time PCR were as follows: RANKL-forward (F) 5′-CAG AAG ATG GCA CTC ACT GCA-3′, RANKL-reverse (R) 5′-CAC CAT CGC TTT CTC TGC TCT-3′; NFATc1-F 5′-AAT AAC ATG CGA GCC ATC ATC-3′, NFATc1-R 5′-TCA CCC TGG TGT TCT TCC TC-3′; CREB1-F 5′-AGC TGC CAC TCA GCC GGG TA-3′, CREB1-R 5′-TGG TGC TCG TGG GTG CTG TG-3′; ATF4-F 5′-GCA TGC TCT GTT TCG AAT GGA-3′; ATF4-R 5′-CCA ACG TGG TCA AGA GCT CAT-3′; GAPDH-F 5′-TCA ATG ACA ACT TTG TCA AGC-3′; and GAPDH-R 5′-CCA GGG TTT CTT ACT CCT TGG-3′. The target genes were normalized to the reference housekeeping gene GAPDH.

### 4.5. Western Blot Analysis and Co-Immunoprecipitation

To prepare samples for Western blot analysis, cells were lysed as previously described [13]. Each sample was subjected to 10% SDS-PAGE and electro-transferred onto PVDF membranes. The membranes were blocked with 5% skimmed milk in TBST, incubated overnight at 4 °C with the relevant primary antibody, followed by incubation with the HRP-conjugated secondary antibody. Immune complexes were visualized using SuperSignal West Pico (Thermo Scientific, Rockford, IL, USA), and chemiluminescence was detected using a MicroChemi device (DNR, Jerusalem, Israel).

To perform immunoprecipitation, cells were co-transfected with NFATc1 and ATF4 expression plasmids. Cells were lysed with the lysis buffer (150 mM NaCl, 25 mM Tris-HCl, 2% Brij35 and 10 mM EDTA supplemented with 1× Complete protease inhibitors) and sonicated briefly. Cell lysate was incubated with 10 µg of NFATc1 antibody, ATF4 antibody, or control IgG, followed by incubation with protein G agarose beads. Bound protein samples were subjected to SDS-PAGE and immunoblot analysis.

### 4.6. Chromatin Immunoprecipitation Assay

The chromatin immunoprecipitation (ChIP) assay was performed according to the protocol previously described [17]. Prepared DNA fragments were immunoprecipitated with NFATc1, ATF4, or CREB1 antibody or control IgG, followed by semi-quantitative or quantitative PCR to amplify the mouse *RANKL* promoter region, including the NFAT-binding element (amplified region: −1070 to −858 bp) and CRE-like element (amplified region: −1147 to −967 bp), with the following primers: NFAT-binding element forward, 5′-GCA AGC TCC AGG CCA GCC TAG-3′, and reverse, 5′-CCA ATA AGA CGG CTC AGC TG-3′ and CRE-like element forward, 5′-AGG AGG CAG AGA TGG CAG AG-3′, and reverse, 5′-ACA CGC GCG CGC GCA AAT A-3′.

### 4.7. Luciferase Reporter Assays

Cells were seeded in a 96-well plate at a density of 1 × 10^4^ cells/well. After overnight culture, the cells were transiently transfected with the reporter plasmids using LipofectAMINE 2000 (Invitrogen, Carlsbad, CA, USA). In each transfection, 0.2 µg of reporters (RANKL-WT, RANKL-MT-N, RANKL-MT-C, NFAT-luc, or pGL3) and *Renilla* luciferase plasmid were used as indicated. The cells were then incubated in the presence of the indicated reagents for 24 h, and luciferase activity was measured using a Dual-Glo luciferase assay kit (Promega, Madison, WI, USA). Each test condition described was represented by three replicate plates. The relative luciferase activity was calculated by dividing firefly luciferase activity by *Renilla* luciferase activity to normalize the transfection efficiency.

### 4.8. Statistical Analysis

Statistical significance was determined using Student’s *t*-test. For the multiple comparisons, one-way analysis of variance was performed. Post hoc analyses for all statistical tests were performed only when significant main effects were detected with the least squares means error test. Differences or changes were considered significant at *p* < 0.05. Luciferase reporter assay was designed with *n* = 6 and other tests were with *n* = 3. For statistical agreements, the number of their repeated experiments was two or three in this study. Data were analyzed using the SAS program (version 9.1; SAS Institute, Cary, NC, USA).
